# ER resident protein 44 promotes malignant phenotype in nasopharyngeal carcinoma through the interaction with ATP citrate lyase

**DOI:** 10.1186/s12967-020-02694-1

**Published:** 2021-02-16

**Authors:** Hui Tian, Si Shi, Bo You, Qicheng Zhang, Miao Gu, Yiwen You

**Affiliations:** 1 Department of Otorhinolaryngology Head and Neck Surgery, Affiliated Hospital of Nantong University, Nantong, Jiangsu China; 2 Institute of Otorhinolaryngology Head and Neck Surgery, Affiliated Hospital of Nantong University, Nantong, Jiangsu China; 3 Medical College of Nantong University, Nantong, Jiangsu China

**Keywords:** ERp44, Nasopharyngeal carcinoma, Metastasis, ACLY, EMT

## Abstract

**Background:**

Nasopharyngeal carcinoma (NPC) is one of the most common malignancy in head and neck. With the development of treatments, the prognosis has improved these years, but metastasis is still the main cause of treatment failure. The endoplasmic reticulum (ER) resident protein 44 is a UPR-induced ER protein of the protein disulphide isomerase (PDI) family. This study investigated the role of ERp44 in NPC progression.

**Methods:**

Firstly, immunohistochemistry, western blot and qRT-PCR were used to investigate the expression of ERp44 in NPC samples and cell lines. We analyzed 44 NPC samples for ERp44 expression and investigated the association between its expression level with clinicopathologic parameters. Then we took CCK8, Transwell migration assay and used the zebrafish model to access the role of ERp44 on the malignant phenotype in NPC cells. Secondly, we used co-IP to gain the proteins that interact with ERp44 and took proteomic analysis. Furthermore, we successfully constructed the mutant variants of ERp44 and found the interaction domain with ATP citrate lyase(ACLY). Lastly, we subcutaneously injected NPC cells into nude mice and took immunohistochemistry to exam the expression of ACLY and ERp44. Then we used western blot to detect the expression level of epithelial-mesenchymal transition (EMT) markers.

**Results:**

In the present study, we found ERp44 was elevated in NPC tissues and correlated with clinical stages and survive state of the patients. In vitro, the downregulation of ERp44 in NPC cells (CNE2, 5-8F) could suppress cells proliferation and migration. After that, we recognized that ACLY might be a potential target that could interact with ERp44. We further constructed the mutant variants of ERp44 and found the interaction domain with ACLY. The promotion of ERp44 on cell migration could be inhibited when ACLY was knocked down. More importantly, we also observed that the interaction of ERp44 with ACLY, especially the thioredoxin region in ERp44 play a vital role in regulating EMT. Lastly, we found ERp44 was positively correlated with the expression of ACLY and could promote NPC cells growth in nude mice.

**Conclusion:**

Our data indicated that ERp44 participates in promoting NPC progression through the interaction with ACLY and regulation of EMT.

## Background

Nasopharyngeal carcinoma (NPC) is one of the most common malignancy in head and neck. According to the International Agency for Research on Cancer (IARC), more than 120,000 new cases were reported in 2018 [1]. It has an unbalanced geographical distribution and > 70% of new cases are reported in east and southeast Asia [2]. The location of the nasopharynx was relatively deep and early symptoms were indistinct. Moreover, NPC has a tendency to invade and metastasize, more than 70% of the patients were locoregionally advanced when they were newly diagnosed [3]. Though the prognosis has improved with the development of treatments, distant metastasis is still the main cause of treatment failure [4, 5]. Therefore, know more about the molecular mechanism of NPC development might help to develop novel prognostic markers and advance treatments for NPC patients.

The endoplasmic reticulum (ER) is a central organelle for proteins to undergo the post-translational modifications to have correct structures [6]. ER is sensitive to cellular stresses, such as low nutrients, poor vascularization and hypoxia, and these pathophysiological conditions could interfere protein folding. In the ER, more and more unfolded proteins accumulate, and unfolded protein response (UPR) is activated [7]. When tumor growth persists, many stresses occur. The activation of UPR results in the increased expression of ER chaperones [8].

The ER resident protein 44(ERp44), a UPR-induced ER protein of the protein disulfide isomerase (PDI) family, was identified as a molecular chaperon of thioredoxin family. The structure of ERp44 contains thioredoxin- and calsequestrin-like domains and a C-terminal ER localization motif [9]. ERp44 controls the localization of key enzymes in the ER and plays an important role in the secretion of correctly assembled disulfide-linked oligomeric proteins [10]. It may regulate protein folding, client protein homeostasis in the ER and Ca^2+^ signaling [11]. With the diverse function of ERp44, it also influences tumor progression. In human oral squamous cancer, ERp44 was highly expressed. After ERp44 was knocked down, the viability of cells was reduced, while apoptosis was significantly induced [12]. In breast cancer, ERp44 was elevated in mammospheres and played key roles in anchorage-independent cell proliferation [13]. Moreover, ERP44 protein was abundant in colorectal cancer(CRC) tissues and could act as a prognostic biomarker [14]. However, there is little evidence of the involvement of ERp44 in NPC progression.

ATP citrate lyase (ACLY) is a lipogenesis enzyme that catalyzes citrate and coenzyme A (CoA) to acetyl-CoA and oxaloacetate. It is reported to be involved in glucose metabolism, lipid synthesis and acetylation reaction [15, 16]. Moreover, ACLY is abnormally expressed in tumors, including myeloid leukemia, gastric adenocarcinoma, glioblastoma and so on [17]. Its expression level is also associated with the poor prognosis of different kinds of tumors [16, 18, 19].

In the present study, we investigated the involvement of ERp44 on NPC malignant phenotype. Firstly, we found that the expression of ERp44 was remarkably higher in both NPC tissues and cell lines. And its overexpression was associated with patients’ survive state and clinical stages. In addition, we performed proteomic analysis and identified ERp44 could interact with ACLY in NPC. Finally, we demonstrated that ERp44-ACLY could regulate NPC metastasis by inducing epithelial-mesenchymal transition(EMT) in vitro and in vivo. All the data suggested that ERp44 participated in promoting malignant phenotype in NPC through, at least in part, the interaction with ACLY and regulation of EMT.

## Methods

### Human NPC specimens and immunohistochemistry

The paraffin-embedded tissues we used in this research were obtained from 44 NPC patients at Affiliated Hospital of Nantong University. Table 1 showed the clinical features of the patients. Fresh samples were maintained at − 80 °C until use. All the patients had not received any anti-tumor treatments at the time of biopsy. We used chronic inflammatory nasopharyngeal epithelium tissues as control. The research was approved by the Ethics Committee of Affiliated Hospital of Nantong University (IRB number: 2018-L049). Every patient knew the propose of the research and the informed consent was got from them.

**Table 1:** The association between ERp44 expression with clinicopathological parameters of NPC

Clinicopathological parameters	Total	ERp44 expression		*p*
		Low	High	
Gender	44			0.851
Male	31	11	20	
Female	13	5	8	
Age (year)				0.598
< 60	27	9	18	
≥ 60	17	7	10	
Clinical stages				0.044*
1	1	1	0	
2	15	9	6	
3	18	4	14	
4	10	2	8	
Survive				0.023*
Death	21	4	17	
Alive	23	12	11	

*Statistical analyses were performed by the Pearson *χ*^2^ test. *p* < 0.05 was considered significant

We analyzed the expression of ERp44 by immunohistochemistry as previously described [20]. 4 μm-thick paraffin-embedded sections were incubated with the antibody of ERp44 (Proteintech,16016–1-AP,1:200). Two pathologists without knowing the patient’s clinicopathological outcomes evaluated the IHC scores according to the intensity of the staining and the percentage of positive cells. The intensity of ERp44 staining was scored as 0, no staining; 1, weak staining; 2, medium staining; 3, strong staining. The percentage of immunopositive cells was scored as 0 < 10; 1,10–25%; 2,26–75%; 3 > 75%. Then the scores were summed and defined as follows: low expression group: 0–3, high expression group:4–6.

### Cell culture

NPC cell lines CNE-1 (high differentiation), CNE-2 (low differentiation), 5-8F (high tumorigenesis and high metastasis), 6-10B (low tumorigenesis and low metastasis) and normal nasopharyngeal epithelial cell line NP69 used in our research were gifted from the Sun Yat-Sen University and Xiang-Ya School of Medicine. NPC cell lines were cultured in RPMI 1640 (Biological Industries Israel Beit-Haemek, 01–100-1ACS) with10% fetal bovine serum (Biological Industries Israel Beit-Haemek, 04–001-1ACS), while NP69 was cultured in Keratinocyte-SFM (Thermo Fisher Scientific, 17005–042). 293T was cultured in DMEM (Biological Industries Israel Beit-Haemek, 06–1055–57-1ACS) with10% fetal bovine serum. All the cells were incubated under the atmosphere at 37 °C in 5% CO^2^ incubator at Otolaryngology Laboratory, Affiliated Hospital of Nantong University.

### Quantitative RT-PCR

qRT-PCR was performed as previously described [20]. We extracted total RNA with Trizol reagent (Thermo Fisher Scientific, 15596018) and reverse transcribed it into cDNA samples using a Transcriptor First Strand cDNA Synthesis Kit (Roche, Germany, 04 896 866 001). We performed the amplification with SYBR Green PCR Master Mix (Roche, 04913914001) in a Real-Time PCR System (Stepone P, Applied Biosystems, Grand Island, NY). The sequences of the primers of ERp44 were as follows: forward: 5′-CCTGTGCCAGGCCTCAATAC -3′, reverse: 5′-TGGCACTGGGCTTCCTGATA -3′. The results were normalized with GAPDH and performed in triplicate.

### Western blotting

Western Blotting was used to access protein expression as previously described [20]. Briefly, we got the proteins with RIPA Lysis Buffer with protease inhibitors. The proteins were electrophoresed by sodium dodecyl sulfate–polyacrylamide gel electrophoresis (SDS-PAGE) with different concentration. Then the proteins were transferred to polyvinylidene difluoride membranes (Millipore, ISEQ00010, Bedford, MA) and blocked in fat-free milk. After that, the membrane was incubated with the primary antibodies including anti-ERp44 (Proteintech, 16016–1-AP,1:500), anti-ACLY (Proteintech, 15421–1-AP,1:500), anti-E-cadherin (Cell Signaling Technology, 1:1000), anti-vimentin (Cell Signaling Technology,1:1000) overnight followed by incubating with HRP-tagged secondary antibody (Santa Cruz Biotechnology,1:1000). Lastly, we visualized the immune complexes with ECL reagent (Millipore).

### Transfection with shRNAs and plasmid

The shRNAs, along with the controls were designed and obtained from Shanghai Genechem Co,Ltd. shERp44-1, forward sequence 5′- GATCCCGCACCCAGTGAATATAGGTATCTCGAGATACCTATATTCACTGGGTGCTTTTTGGAT-3′, shERp44-2, forward sequence 5′- GATCCCGCTCGGCAATTAATAAGTGAACTCGAGTTCACTTATTAATTGCCGAGCTTTTTGGAT-3′, shERp44-3, forward sequence 5′- GATCCCCCGATGTCATTAAGGAAGAATCTCGAGATTCTTCCTTAATGACATCGGTTTTTGGAT-3′. The plasmids containing tag HA were constructed by Guangzhou GeneCopoeia Co,Ltd. They were wild type ERp44 (ERp44-wt), wild type ACLY (ACLY-wt), mutations in the thioredoxin region (ERp44 Δ1, mutant in 30–138), mutations in other than thioredoxin region (ERp44 Δ 2, mutant in 139–402). We did the transfection with Lipofectamine 2000 (Invitrogen, USA) according to the manufacturer’s instructions.

### CCK8 assay

CNE2 or 5-8F cells transfected with ERp44-shRNA or control were seeded into a 96-well plate (Corning inc, Corning NY) at a density of 1 × 10^4^ cells per well. CCK8 assay (Beyotime Institute of Biotechnology, China) was used to access cell proliferation at time points of 0, 6, 12, 24, 36 h. The absorbance was accessed with a microplate reader (F-2500 Fluorescence Spectro-photometer; Hitachi) at 450 nm.

### Transwell migration assay

Millipore chambers containing a polycarbonate flitter of 8 mm (Millipore) were used to measure cell migration. 5 × 10^4^ Cells treated with shRNA or control were resuspended in 200 ul serum-free medium and seeded on the upper chamber, while culture medium with 10% FBS was added to the lower chamber. 16 h later, we removed the cells in the upper surface and fixed the cells adhering on the lower surface in 100% methanol. Then we stained the cells with 0.1% crystal violet. The migration cells were visualized under a microscope.

### Wound-healing assay

CNE2 or 5-8F cells were seeded on 6-well plates and cotransfected with wild-type plasmids and shRNAs. When cells confluence reached about 80%, we made scratches with a 100 ul pipette tip. Then the 6-well plates were incubated at 37 °C. At different time points, the migration distance of cells was imaged under a microscope. The relative migrated width was calculated by the wound width/the distance measured at 0 h.

### Colony-formation assay

50 cells which had transfected with ERp44-shRNA or control were seeded on 6-well plates. 14 days later, we washed the plate twice in 1 × PBS and fixed the cells in 4% paraformaldehyde for 30 min. After that, we stained the cells with 0.1% crystal violet solution for 30 min. Colonies that contain more than 50 cells was counted under the microscope.

### Co-IP

Co-IP was performed according to the instructions of Pierce Co-Immunoprecipitation Kit (Thermo Scientific, 26149). Briefly, we performed all co-IP steps at 4 °C. We washed the resin twice by adding 200μL of IP Lysis/Wash Buffer to the spin column containing the antibody-coupled resin, then centrifuged and discarded the flow-through. After that, we gently tapped the bottom of the spin column on a paper towel to remove excess liquid and inserted the bottom plug. Then it was incubated with gentle mixing or rocking overnight at 4 °C. The spin columns were centrifuged and the flow-through was saved for future analysis. We placed the column into a new tube, added 200μL of IP Lysis/Wash Buffer and centrifuged. Lastly, the pellets were identified by western blot with antibodies at the following dilutions: anti-ERp44 (Proteintech, 16016–1-AP,1:500), anti-ACLY(Proteintech, 15421–1-AP,1:500), anti-HA-tag (Sangon Biotech, 1:1000).

### Zebrafish metastasis model

We used transgenic zebrafish *Tg (fli1a: EGFP)* to observe tumor migration. The research was conducted in Zebrafish Center at Nantong University under standard conditions as previously described [21]. We collected fertilized zebrafish eggs at 28 °C and treated 22 h post fertilization (hpf) embryos with 0.2 mM 1-phenyl-2-thiourea to prevent pigmentation. The xenotransplantations were performed in 48 hpf zebrafish embryos. We labeled tumor cells with 2 g/ml of DiI (Fluka, Germany) and approximately 100–500/5 nl tumor cells were injected into the perivitelline cavity of each embryo using a microinjection system (WPI). We visualized the cells that disseminated from primary sites under the fluorescence microscope and 5 random fields were observed in each chamber.

### Animal xenograft tumor model

To assess the role of ERp44 on NPC progression in vivo, we obtained 5-week-old male BALB/c mice from the Laboratory Animal Center of Nantong University. 1 × 10^6^ CNE2 cells transfected with shERp44 or control were subcutaneously injected into the mice. Tumors were measured every two days and the volume was calculated as (length × width^2^)/2. Three weeks later, tumor tissues were obtained and fixed in 10% formalin or kept at – 80 °C for further research. We used IHC to detect the expression of ERp44 and ACLY. The intensity of tumor staining was scored as 0, no staining;1, weak staining;2, medium staining;3, strong staining. The percentage of immunopositive cells were scored as 0, < 10%; 1, 10–25%; 2,26–75%; 3, > 75%. Then we summed the scores, we defined the expression level of ERp44 and ACLY as follows: “1” (0), “2” (1, 2), “3” (3, 4), “4” (5, 6). Studies in animals were approved by the committee on the Ethics of Animal Experiments of Nantong University (RDD number:20180227–008). The experiments were followed NIH Guidelines and were approved by the Administration Committee of Experimental Animals, Jiangsu Province, China (Approval ID: SYXK(SU)2007–0021).

### Statistical analysis

All the experiments were performed 3 times and the data were presented as mean ± standard deviation (SD). We analyzed the data using GraphPad Prism 6 and SPSS17.0 software. The associations among ERp44 expression with clinicopathological features were analyzed using the *χ*^2^ test. Two-tailed student’s t-tests were used to determine statistical significance. *p* < 0.05 was considered statistically significant.

## Results

### ERp44 was highly expressed in NPC

**Fig. 1:**
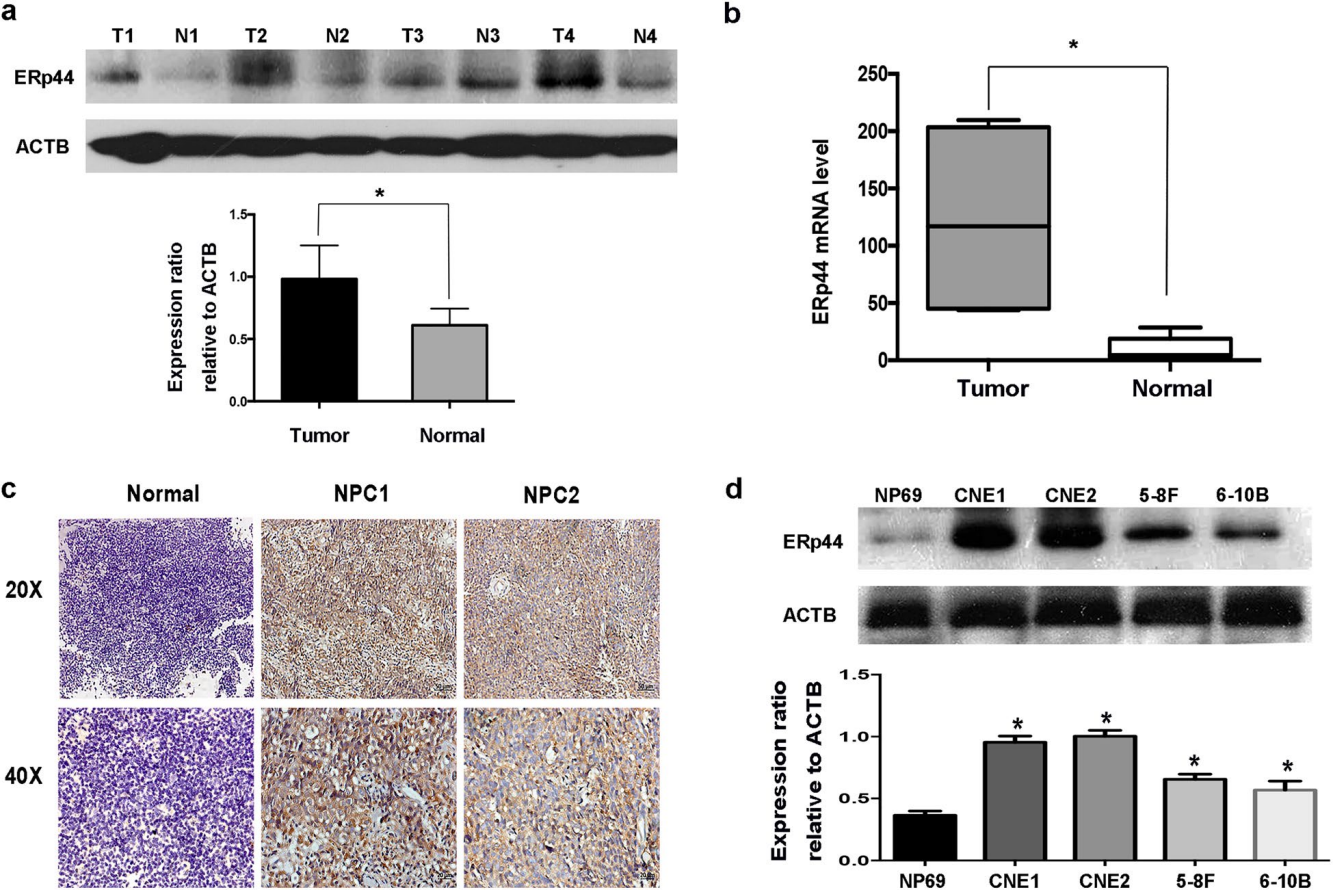
ERp44 was highly expressed in NPC. **a** Western blot analysis was performed to reveal ERp44 expression in tissues. Tumor: Nasopharyngeal squamous cell carcinoma tissues. Normal: Inflammatory nasopharyngeal epithelium tissues. ACTB was used as a control. The bar demonstrated the expression of ERp44 relative to ACTB by densitometry. **b** qRT-PCR was used to detect ERp44 expression in NPC tissues and inflammatory nasopharyngeal tissues. **c** Representative results of immunohistochemical staining. Left: Inflammatory nasopharyngeal epithelium tissues had lower expression of ERp44 (up: × 20) (down: × 40). Middle and Right Line: NPC tissues had higher expression of ERp44 (up: × 20) (down: × 40). **d** Western blot was used to detect ERp44 expression in NPC cell lines CNE1, CNE2, 5-8F, 6-10B and normal nasopharyngeal epithelial cell NP69. Data represent mean ± SEM. *p < 0.05

In order to investigate the function of ERp44 in NPC, first of all, we performed western blot and qRT-PCR to compare the expression level of ERp44 in NPC tissues with that in normal tissues in the nasopharynx. The results showed that both the protein and mRNA levels of ERp44 were increased in most NPC tissues (Fig. 1a, b). To further confirm our results, IHC analysis was used to explore the expression and subcellular localization of ERp44 in NPC specimens. We found in most NPC samples, ERp44 was cytoplasmic positively staining, and more importantly, it had higher expression in NPC than normal tissues (Fig. 1c). Besides that, compared with normal cell line NP69, ERp44 was dramatically increased in NPC cell lines (CNE1, CNE2, 5-8F, 6-10B) (Fig. 1d). These results suggested that ERp44 was highly expressed in NPC.

### Clinical significance of ERp44 in NPC

We then analyzed the relationship between ERp44 expression with NPC clinicopathologic variables. The data from IHC showed that ERp44 expression level was significantly associated with clinical stages (*p* < 0.05) and the survive state of NPC patients (*p* < 0.05). However, it did not correlate with gender and age of the patients (*p* > 0.05) (Table 1).

### Interference of ERp44 expression could inhibit the malignant phenotype of NPC cells

**Fig. 2:**
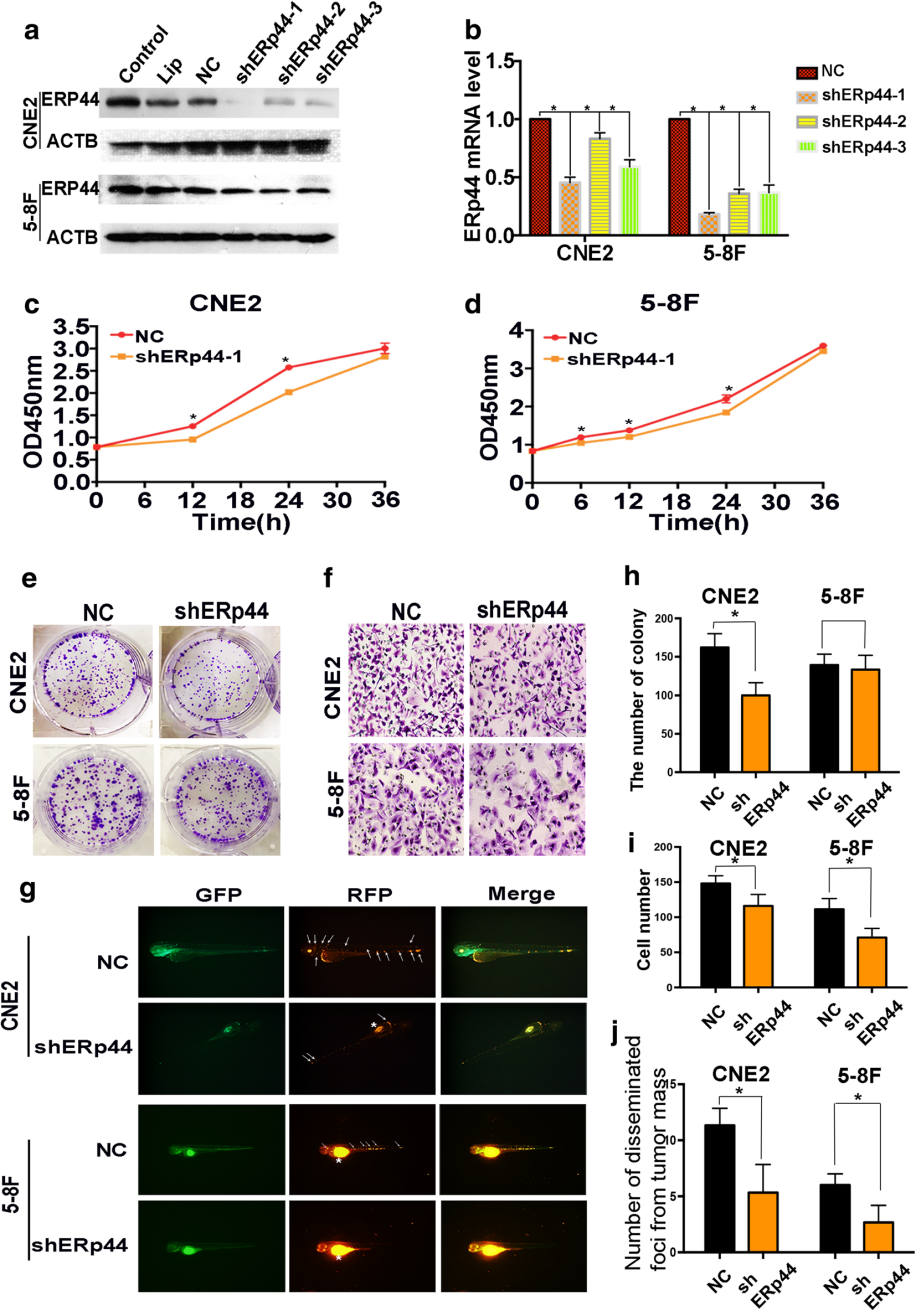
Interference of ERp44 expression could inhibit the malignant phenotype of NPC cells. **a** CNE2 and 5-8F cells were transfected with shRNA targeting ERp44 (sh-1, sh-2, sh-3) or a scrambled sequence (NC). **b** qRT-PCR was used to detect the mRNA level of ERp44 after the transfection. **c**, **d** CCK8 was used to determine cell proliferation after the treatment of shERp44 in CNE2 and 5-8F. **e**, **h** Knockdown of ERp44 in CNE2 could significantly reduce colony formation. We showed the representative images and the quantification analysis. **f**, **i** The cells migrated through the membrane in a transwell after ERp44 was knocked down. We showed the representative images of the migrated cells. **g**, **j** *Tg(fli1a: EGFP)* transgenic zebrafish was used to detect cell metastasis. Tumor cells were stained in red. The asterisks represented tumor cells in primary sites. The arrows represented tumor cells in disseminated foci. We observed the number of disseminated foci from primary sites after cells were treated with sh-ERp44. All experiments were repeated three times. Data represent mean ± SEM. *p < 0.05

Due to the expression pattern of ERp44 in NPC, we therefore investigated whether it took a role in the malignant phenotype of NPC cells. As CNE2 is the most common pathological pattern of NPC and 5-8F has characteristic of high tumorigenesis and high metastasis, we chose CNE2 and 5-8F for further studies. Transfection was preformed to downregulate ERp44 in NPC cells. The knockdown efficiency was confirmed by western blot and qRT-PCR, we found shERp44-1 was the most efficiency, so we took it for further studies (Fig. 2a, b). CCK8 assay showed that cell proliferation was inhibited when ERp44 was knocked down (Fig. 2c, d). Besides that, cells treated with shERp44 had lower capability of colony formation in CNE2 cells (Fig. 2e, h). So ERp44 could inhibit the growth of NPC cells in vitro.

As metastasis was the most important feature of NPC cells, we then detect the role of ERp44 on cell migration. Transwell migration assay showed that suppressing ERp44 expression could inhibit cell migration (Fig. 2f, i). Zebrafish has been reported to have advantages in tumor research [22], and we used zebrafish model to observe tumor metastasis. We injected NPC cells with different treatments into the perivitelline cavity of the *Tg(fli1a: EGFP*) transgenic zebrafish and observed the process of tumor cell metastasis. 3 days after implantation, ERp44-shRNA treated CNE2 and 5-8F cells had less number of disseminated foci from primary sites than negative control group (Fig. 2g, j). So ERp44 might promote NPC progression by accelerating cells migration. In a word, ERp44 could promote the malignant phenotype of NPC cells.

### ERp44 could interact with ACLY in NPC cells

**Fig. 3:**
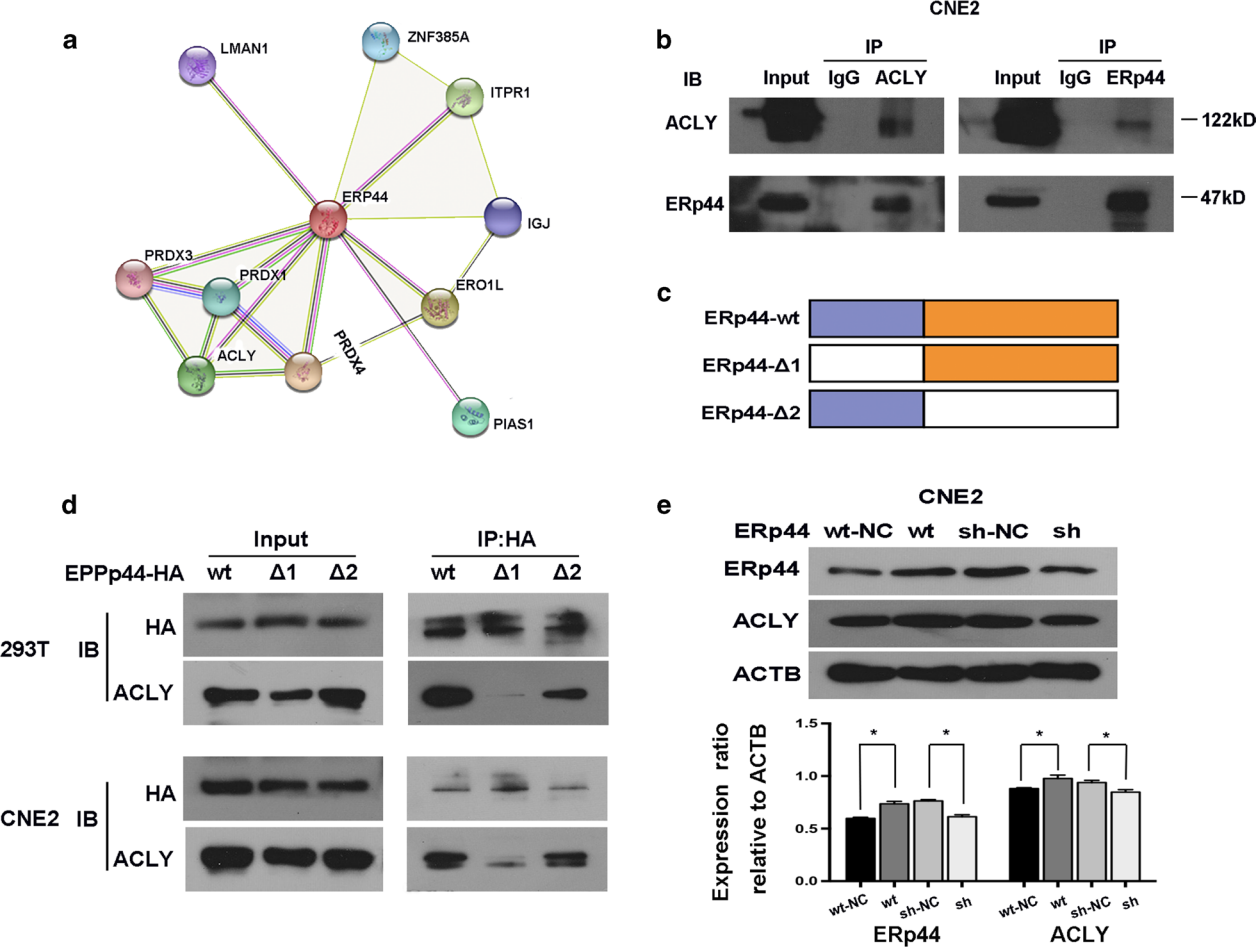
ERp44 could interact with ACLY in NPC cells. **a** The results from string protein interaction network, it showed the proper proteins that might interact with ERp44. **b** Co-IP was used to confirm the interaction of ERp44 with ACLY. **c** The mutant variants of ERp44 containing the tag HA, mutations in the thioredoxin region (ERp44-Δ 1, mutant in 30–138), mutations in other than thioredoxin region (ERp44-Δ 2, mutant in 139–402). **d** Co-IP confirmed that ERp44-Δ1was the interaction region with ACLY in 293 T and CNE2 cells. **e** Western blot showed that when ERp44 was knockdown, there was similar tread of expression change in ACLY. All experiments were repeated three times. Data represent mean ± SEM. *p < 0.05

The results above caught our interest to further investigate the internal mechanism. Firstly, we search string protein interaction network (https://version-10-5.string-db.org/) to predict the potential proteins that could interact with ERp44. Then we did co-IP to pull down the proteins and took proteomic analysis. Gene Ontology (GO) enrichment analysis showed that the genes were associated with cell adherent, cell differentiation and cell apoptosis. After that, we analyzed the results and recognized that ACLY might be a potential target that could interact with ERp44 (Fig. 3a). The interaction between ERp44 and ACLY was detected by immunoprecipitation. In accordance with our hypothesis, ERp44 and ACLY were interacted in CNE2 cells (Fig. 3b). Furthermore, we identified the region within ERp44 that take a role in ERp44-ACLY interaction. We successfully constructed two mutant variants of ERp44 and designed the plasmids containing tag HA. They were wild type ERp44 (ERp44-wt), mutations in thioredoxin region (ERp44 Δ1, mutant in 30–138), mutations in other than thioredoxin region (ERp44 Δ 2, mutant in 139–402). As showed in Fig. 3c, d, we demonstrated that, both in 293 T and CNE2, ERp44 Δ 1was the interaction region with ACLY. Moreover, we found that when the expression of ERp44 was knocked down, there was similar tread of expression change in ACLY (Fig. 3e). So ERp44 could interact with ACLY in NPC cells.

### The binding between ERp44 with ACLY was critical for ERp44-mediated regulation of NPC metastasis

**Fig. 4:**
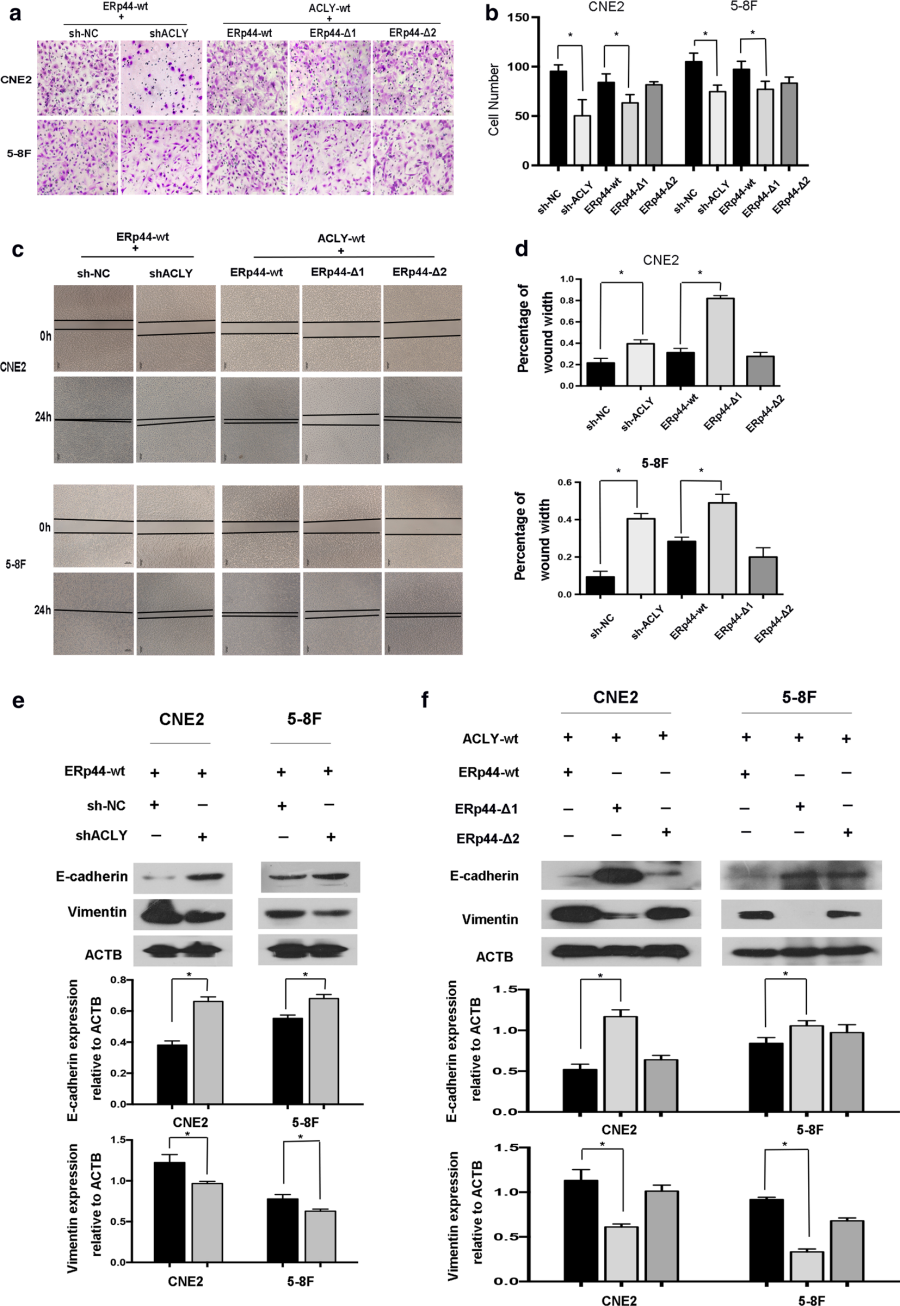
The binding between ERp44 with ACLY was critical for ERp44-mediated regulation of NPC metastasis. **a**, **b** Transwell migration assay showed that downregulating the expression of ACLY could inhibit the migration of NPC cells. The migrated cells were less in ACLY-wt cells co-transfected with ERp44 Δ1 cells compared with that in wild-type cells or Δ2 mutant cells. **c**, **d** Wound-healing assay showed cells treated with ERp44 Δ1 migrated slower than other two groups. Representative images of cells migration were shown at 0 and 24 h with a microscope. The relative migrated width was calculated by the wound width/the distance measured at 0 h. The histogram showed the relative distance of wound. **e** Western blot showed that when ACLY was downregulated, E-cadherin was increased, whereas vimentin was reduced. The histogram showed the expression of E-cadherin or Vimentin relative to ACTB. **f** Western blot showed that when co-transfected ACLY-wt cells with wild- type ERp44, ERp44 Δ1or ERp44 Δ2 mutant cells, only ERp44 Δ1 could increase the expression of E-cadherin and decrease Vimentin. The histogram showed the expression of proteins relative to ACTB. All experiments were repeated three times. Data represent mean ± SEM. *p < 0.05

We next investigated the significance of ERp44-ACLY in NPC cells. Transwell migration assay and wound-healing assay showed that downregulating the expression of ACLY could inhibit the migration of CNE2 and 5-8F (Fig. 4a, c). Then, we analyzed the effects of ERp44 binding to ACLY in regulating the metastasis of NPC cells. Cell metastasis was accessed in ACLY-wt NPC cells re-expressing wild-type ERp44, ERp44 Δ1or Δ2 mutant cells. The results showed that migrated cells were less in ERp44 Δ1 cells compared with that in wild-type cells or Δ2 mutant cells (Fig. 4a, c). In a word, the interaction of ERp44 with ACLY, especially the thioredoxin region in ERp44 play a vital role in NPC metastasis.

### ERp44 promoted NPC metastasis through the promotion of EMT

As we know, EMT is a process that play important roles in tumor metastasis, invasion, and chemoresistance [23]. In our research, we found that when ACLY was downregulated, the progress of EMT was inhibited. As shown in Fig. 4e, E-cadherin was increased, whereas Vimentin was reduced. To further confirm the role of ERp44 Δ1 on EMT progress, we co-transfected ACLY-wt cells with wild- type ERp44, ERp44 Δ1or ERp44 Δ2 mutant cells. The results showed that both in CNE2 and 5-8F, only ERp44 Δ1 could increase the expression of E-cadherin and decrease Vimentin (Fig. 4f). Taken together, ERp44 could interact with ACLY to regulate cell migration via EMT.

### ERp44 promoted NPC cells growth in vivo

**Fig. 5:**
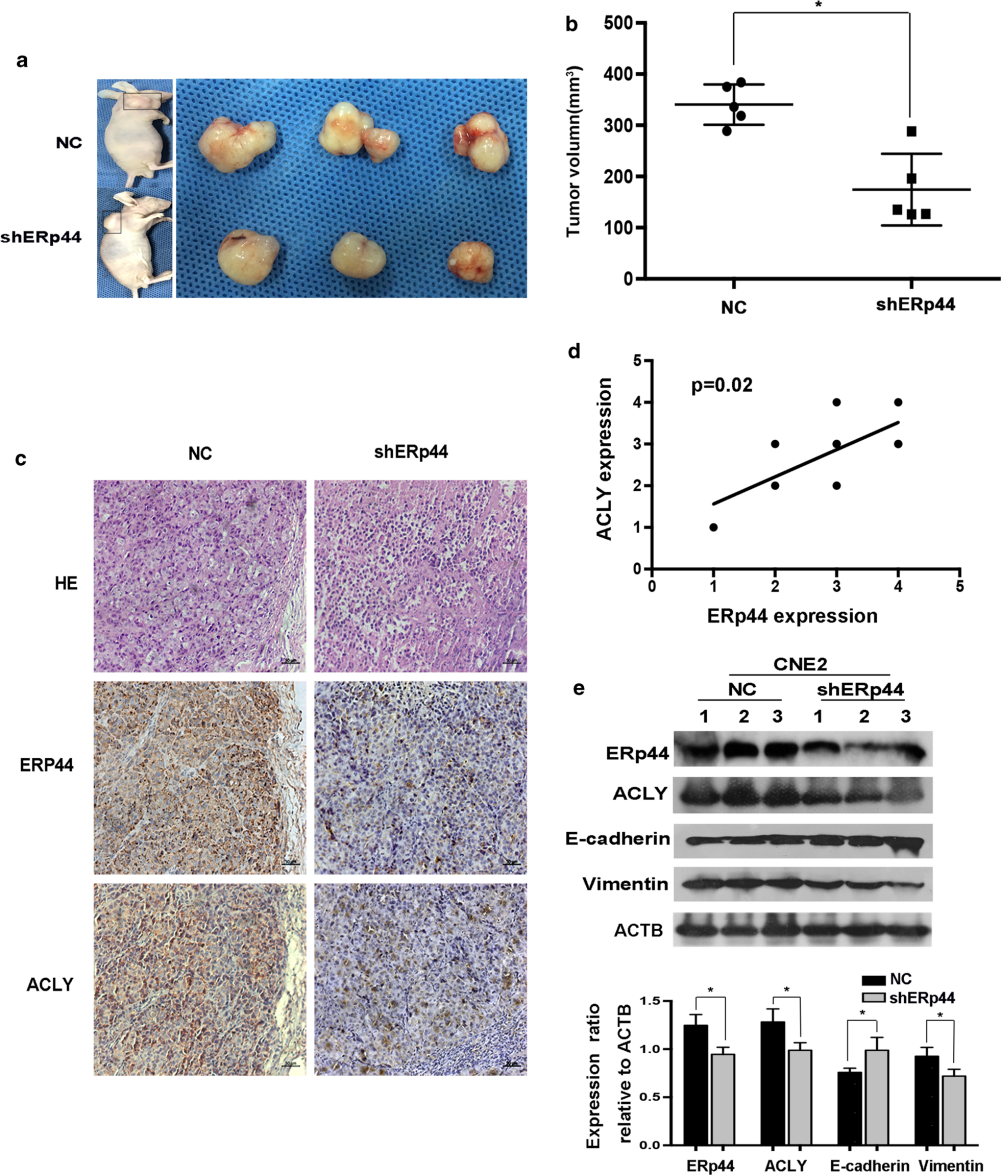
ERp44 promoted NPC cells growth in vivo. **a** Nude mice were subcutaneously injected with CNE2 cells transfected with NC or shERp44. We showed the representative pictures of NPC xenografts in nude mice and tumors. **b** The volume of tumors treated with shERp44 were smaller than control (n = 5 per group). **c** Immunohistochemistry was used to access ERp44 and ACLY expression in xenografts, we showed the representative pictures. **d** The correlation of ERp44 with ACLY expression in xenografts derived from nude mice. **e** Western blot showed E-cadherin was upregulated in shERp44 tumor sections, while Vimentin was downregulated. Experiments were repeated three times. Data represent mean ± SEM. *p < 0.05

To further corroborate the role of ERp44 on NPC tumorigenicity, we subcutaneously injected CNE2 cells that had been transfected with shERp44 or control into nude mice. As expected, when ERp44 was downregulated, tumor growth was inhibited (Fig. 5a). The volume of tumors treated with shERp44 were also smaller than control (Fig. 5b). Then we took immunohistochemistry to access the expression of ACLY and ERp44. We found when ERp44 was downregulated, ACLY expression was also decreased. Moreover, the level of ERp44 was positively correlated with the expression of ACLY (Fig. 5c, d). Western blot showed that E-cadherin was upregulated in shERp44 tumor sections, while Vimentin was downregulated (Fig. 5e). These data suggested that ERp44 could promote NPC cells growth in nude mice.

## Disscusion

Tumor progression is a complicated process and hundreds of molecules participate in it [24]. NPC has a high tendency to metastasize. Early metastasis and local invasion might be important features of NPC [25, 26]. With the development of radiotherapy and chemotherapy, local control of NPC has improved, but metastasis is still the main cause of poor prognosis [4, 27]. Here, we have shown that ERp44 was associated with the malignant phenotype of NPC and participated in promoting NPC development  through the interaction with ACLY and regulation of EMT.

ERS plays important roles in tumor development. It is prevalent in tumor tissues and is involved in tumor progression [6, 28]. ER resident protein 44 is induced during ER stress and is an ER resident thioredoxin (TRX)-like motif-containing protein. Studies have reported the abnormal expression of ERp44 in tumor. However, the expression of ERp44 in NPC remain poorly understood.

One of the most important findings of this research was that ERp44 had higher expression level in NPC than normal tissues (Fig. 1a–c). Moreover, we found that it was significantly associated with clinical stages and the survive state of NPC patients (*p* < 0.05). Our results were consistent with the previous research. María Garranzo-Asensio et al. took the in-depth seroproteomic analysis of colorectal cancer and identified ERp44 could discriminate the metastasis and recurrence of patients. It could act as a valuable biomarker with diagnostic and prognostic ability [14]. We therefore hypothesized that high level of ERp44 might play an important role in the progression of NPC.

Next, we focused on whether ERp44 took effects on cell malignant phenotype. CCK8, colony formation and Transwell migration assay showed that ERp44 could promote cell proliferation and migration (Fig. 2). Studies on the function of ERp44 on tumor diverse. In breast cancer, ERp44 played an important role in anchorage-independent cell proliferation [13]. On the other hand, Xue Huang et al. reported that ERp44 inhibited A549 cell migration, and this effect might be associated with its regulation on intracellular calcium activities via an inositol 1,4,5-trisphosphate receptor type 2 (IP3R2)-dependent pathway, which was involved in regulating cell migration [29]. The diverse functions of ERp44 might depend on its interactions with various clients. We further investigated the mechanism by which ERp44 affected the malignant biological behavior of NPC cells.

ERp44, a landmark protein of ER stress, contains a thioredoxin(TRX) region and is induced during ER stress. Chihiro Hisatsune et al. reported that ERp44 could interact with ER aminopeptidase I(ERAP1) and control its localization to regulate blood pressure. Previous studies have reported that there are two cysteines in the trx-like domain (C29 and C63). They also suggested C29 of ERp44 was a major binding site for ERAP1 [10, 30]. Higo et al. reported that ERp44 could bind to IP3R1, and regulate intracellular Ca^2+^ concentrations by modulating IP3R1 activity [31]. Moreover, Pan J et al. found C160/212 of L3V domain could take effect on the binding of IP3R1 and ERp44 [32].

In our research, we searched the string protein interaction network and predicted that ERp44 might interact with PRDX4, Ero1-L, ITPR1, ACLY, PIAS1, PRDX1, ZNF-385A, IGJ, LMAN1, PRDX3 (Fig. 3a). Then, we combined our results from proteomic analysis with the data from String, and further reduced the scope. Co-IP was used to confirm our hypothesis. Our results showed that ERp44 could interact with ACLY (Fig. 3b).

More and more evidences highlight the importance of ACLY in tumor. It could regulate tumor metabolism by regulating glucose catabolism, protein acetylation, lipid and nucleotide synthesis [33]. Aberrantly expression of ACLY was found in many types of tumors [16]. In gastric adenocarcinoma, high ACLY expression level was associated with the advanced stage and poor prognosis [18]. In hepatocellular carcinoma, knockdown of ACLY could suppress cells migration and invasion [34]. Similarly, in our study, we also found that after ACLY was knocked down, the migration of tumor cells was inhibited (Fig. 4a, c).

In order to clarify the interaction region between ERp44 and ACLY, we mutated the structural domain of ERp44. Firstly, we successfully constructed the mutant variants of ERp44 and designed the plasmids containing tag HA. Then, Co-IP was preformed to detect the interaction between ACLY and ERp44 variants. The results showed that when we mutated the thioredoxin region in ERp44, there was less interaction happened. So the thioredoxin region in ERp44 was the interaction domain with ACLY (Fig. 3c, d). Moreover, wound-healing assay and Transwell migration assay showed that the migrated cells were less in ERp44 Δ1 cells compared with that in wild-type cells or Δ2 mutant cells (Fig. 4a–d).

The process of tumor metastasis is complex and tumor cells acquire migration and invasion ability to diffuse into the distant places [35]. During this process, EMT plays important roles. It occurs accompanied with the loss of epithelial markers the gain of mesenchymal markers [36]. Studies have reported that some oncogenes could decrease the expression of E-cadherin and induce mesenchymal phenotypes to promote NPC metastasis [37, 38]. In colon cancer, ACLY was highly expressed and could promote cell metastasis, especially the process of EMT [39]. In our research, we found that when ACLY was downregulated, the progress of EMT was inhibited, and ERp44 Δ1 could increase the expression of E-cadherin and decrease Vimentin. More importantly, we also confirmed the role of ERp44 in vivo (Fig. 5). All the data suggested that ERp44 could interact with ACLY to promote malignant phenotype of NPC cells via EMT.

## Conclusions

Our research provided evidence that ERp44 was associated with the progression of NPC and participated in promoting malignant phenotype of NPC cells through the interaction with ACLY and regulation of EMT. These results suggested that the activation of ERp44 might be an important factor for driving NPC progression and it could be considered a potential target for therapy.

## Data Availability

All data generated during this study are included in this published article.

## Abbreviations

NPC: Nasopharyngeal carcinoma
IARC: International Agency for Research on Cancer
ER: Endoplasmic reticulum
ERP44: Endoplasmic reticulum resident protein 44
PDI: Protein disulphide isomerase
UPR: Unfolded protein response
TRX: Thioredoxin
ACLY: ATP citrate lyase
EMT: Epithelial-mesenchymal transition
GO: Gene Ontology
CoA: Coenzyme A
IHC: Immunohistochemistry
SDS-PAGE: Sodium dodecyl sulfate–polyacrylamide gel electrophoresis

## References

[1] Bray F, Ferlay J, Soerjomataram I, Siegel RL, Torre LA, Jemal A (2018). Global cancer statistics 2018: GLOBOCAN estimates of incidence and mortality worldwide for 36 cancers in 185 countries. CA Cancer J Clin.

[2] Chen YP, Chan ATC, Le QT, Blanchard P, Sun Y, Ma J (2019). Nasopharyngeal carcinoma. Lancet (London, England).

[3] Sun Y, Li WF, Chen NY, Zhang N, Hu GQ, Xie FY (2016). Induction chemotherapy plus concurrent chemoradiotherapy versus concurrent chemoradiotherapy alone in locoregionally advanced nasopharyngeal carcinoma: a phase 3, multicentre, randomised controlled trial. Lancet Oncol.

[4] Peng LX, Wang MD, Xie P, Yang JP, Sun R, Zheng LS (2020). LACTB promotes metastasis of nasopharyngeal carcinoma via activation of ERBB3/EGFR-ERK signaling resulting in unfavorable patient survival. Cancer Lett.

[5] Liang YY, Deng XB, Lin XT, Jiang LL, Huang XT, Mo ZW (2020). RASSF1A inhibits PDGFB-driven malignant phenotypes of nasopharyngeal carcinoma cells in a YAP1-dependent manner. Cell Death Dis.

[6] Han CC, Wan FS (2018). New insights into the role of endoplasmic reticulum stress in breast cancer metastasis. J Breast Cancer.

[7] Siwecka N, Rozpędek W, Pytel D, Wawrzynkiewicz A, Dziki A, Dziki Ł (2019). Dual role of endoplasmic reticulum stress-mediated unfolded protein response signaling pathway in carcinogenesis. Int J Mol Sci..

[8] Chang Y, Wu Y, Liu W, Ji G (2015). Knockdown of ERp44 leads to apoptosis via activation of ER stress in HeLa cells. Biochem Biophys Res Commun.

[9] Anelli T, Alessio M, Mezghrani A, Simmen T, Talamo F, Bachi A (2002). ERp44, a novel endoplasmic reticulum folding assistant of the thioredoxin family. EMBO J.

[10] Tempio T, Anelli T (2020). The pivotal role of ERp44 in patrolling protein secretion. J Cell Sci.

[11] Yang K, Li DF, Wang X, Liang J, Sitia R, Wang CC (2016). Crystal structure of the ERp44-peroxiredoxin 4 complex reveals the molecular mechanisms of thiol-mediated protein retention. Structure.

[12] Cho JH, Jeon YJ, Park SM, Shin JC, Lee TH, Jung S (2015). Multifunctional effects of honokiol as an anti-inflammatory and anti-cancer drug in human oral squamous cancer cells and xenograft. Biomaterials.

[13] Wise R, Duhachek-Muggy S, Qi Y, Zolkiewski M, Zolkiewska A (2016). Protein disulfide isomerases in the endoplasmic reticulum promote anchorage-independent growth of breast cancer cells. Breast Cancer Res Treat.

[14] Garranzo-Asensio M, San Segundo-Acosta P, Povés C, Fernández-Aceñero MJ, Martínez-Useros J, Montero-Calle A (2020). Identification of tumor-associated antigens with diagnostic ability of colorectal cancer by in-depth immunomic and seroproteomic analysis. J Proteomics.

[15] Fernandez S, Viola JM, Torres A, Wallace M, Trefely S, Zhao S (2019). Adipocyte ACLY facilitates dietary carbohydrate handling to maintain metabolic homeostasis in females. Cell Rep.

[16] Granchi C (2018). ATP citrate lyase (ACLY) inhibitors: An anti-cancer strategy at the crossroads of glucose and lipid metabolism. Eur J Med Chem.

[17] Lee JV, Berry CT, Kim K, Sen P, Kim T, Carrer A (2018). Acetyl-CoA promotes glioblastoma cell adhesion and migration through Ca(2+)-NFAT signaling. Genes Dev.

[18] Qian X, Hu J, Zhao J, Chen H (2015). ATP citrate lyase expression is associated with advanced stage and prognosis in gastric adenocarcinoma. Int J Clin Exp Med.

[19] Wang J, Ye W, Yan X, Guo Q, Ma Q, Lin F (2019). Low expression of ACLY associates with favorable prognosis in acute myeloid leukemia. J Transl Med.

[20] Shi S, Cao X, Gu M, You B, Shan Y, You Y (2015). Upregulated expression of SOX4 is associated with tumor growth and metastasis in nasopharyngeal carcinoma. Dis Markers.

[21] Huang Y, Wang X, Wang X, Xu M, Liu M, Liu D (2013). Nonmuscle myosin II-B (myh10) expression analysis during zebrafish embryonic development. Gene Expr Patterns.

[22] Shan Y, You B, Shi S, Shi W, Zhang Z, Zhang Q (2018). Hypoxia-induced matrix metalloproteinase-13 expression in exosomes from nasopharyngeal carcinoma enhances metastases. Cell Death Dis.

[23] Aiello NM, Maddipati R, Norgard RJ, Balli D, Li J, Yuan S (2018). EMT subtype influences epithelial plasticity and mode of cell migration. Dev Cell..

[24] Qian CN, Mei Y, Zhang J (2017). Cancer metastasis: issues and challenges. Chin J Cancer.

[25] Fu ZT, Guo XL, Zhang SW, Zeng HM, Sun KX, Chen WQ (2018). Incidence and mortality of nasopharyngeal carcinoma in China, 2014. Zhonghua Zhong Liu Za Zhi.

[26] Guo J, Ma J, Zhao G, Li G, Fu Y, Luo Y (2017). Long noncoding RNA LINC0086 functions as a tumor suppressor in nasopharyngeal carcinoma by targeting miR-214. Oncol Res.

[27] Guo Q, Lu T, Chen Y, Su Y, Zheng Y, Chen Z (2016). Genetic variations in the PI3K-PTEN-AKT-mTOR pathway are associated with distant metastasis in nasopharyngeal carcinoma patients treated with intensity-modulated radiation therapy. Sci Rep.

[28] Bahar E, Kim JY, Yoon H (2019). Chemotherapy resistance explained through endoplasmic reticulum stress-dependent signaling. Cancers (Basel).

[29] Huang X, Jin M, Chen YX, Wang J, Zhai K, Chang Y (2016). ERP44 inhibits human lung cancer cell migration mainly via IP3R2. Aging.

[30] Hisatsune C, Ebisui E, Usui M, Ogawa N, Suzuki A, Mataga N (2015). ERp44 exerts redox-dependent control of blood pressure at the ER. Mol Cell.

[31] Higo T, Hattori M, Nakamura T, Natsume T, Michikawa T, Mikoshiba K (2005). Subtype-specific and ER lumenal environment-dependent regulation of inositol 1,4,5-trisphosphate receptor type 1 by ERp44. Cell.

[32] Pan C, Zheng J, Wu Y, Chen Y, Wang L, Zhou Z (2011). ERp44 C160S/C212S mutants regulate IP3R1 channel activity. Protein Cell.

[33] Icard P, Wu Z, Fournel L, Coquerel A, Lincet H, Alifano M (2020). ATP citrate lyase: a central metabolic enzyme in cancer. Cancer Lett.

[34] Han Q, Chen CA, Yang W, Liang D, Lv HW, Lv GS (2020). ATP-citrate lyase regulates stemness and metastasis in hepatocellular carcinoma via the Wnt/β-catenin signaling pathway. HBPD Int.

[35] Liu Y, Cao X (2016). Characteristics and Significance of the Pre-metastatic Niche. Cancer Cell.

[36] Lamouille S, Xu J, Derynck R (2014). Molecular mechanisms of epithelial-mesenchymal transition. Nat Rev Mol Cell Biol.

[37] Yang Z, Wang J, Zhang Z, Tang F (2019). Epstein-barr virus-encoded products promote circulating tumor cell generation: a novel mechanism of nasopharyngeal carcinoma metastasis. OncoTargets Ther.

[38] Yang XZ, Chen XM, Zeng LS, Deng J, Ma L, Jin C (2020). Rab1A promotes cancer metastasis and radioresistance through activating GSK-3β/Wnt/β-catenin signaling in nasopharyngeal carcinoma. Aging.

[39] Wen J, Min X, Shen M, Hua Q, Han Y, Zhao L (2019). ACLY facilitates colon cancer cell metastasis by CTNNB1. J Exp Clin Cancer Res.

